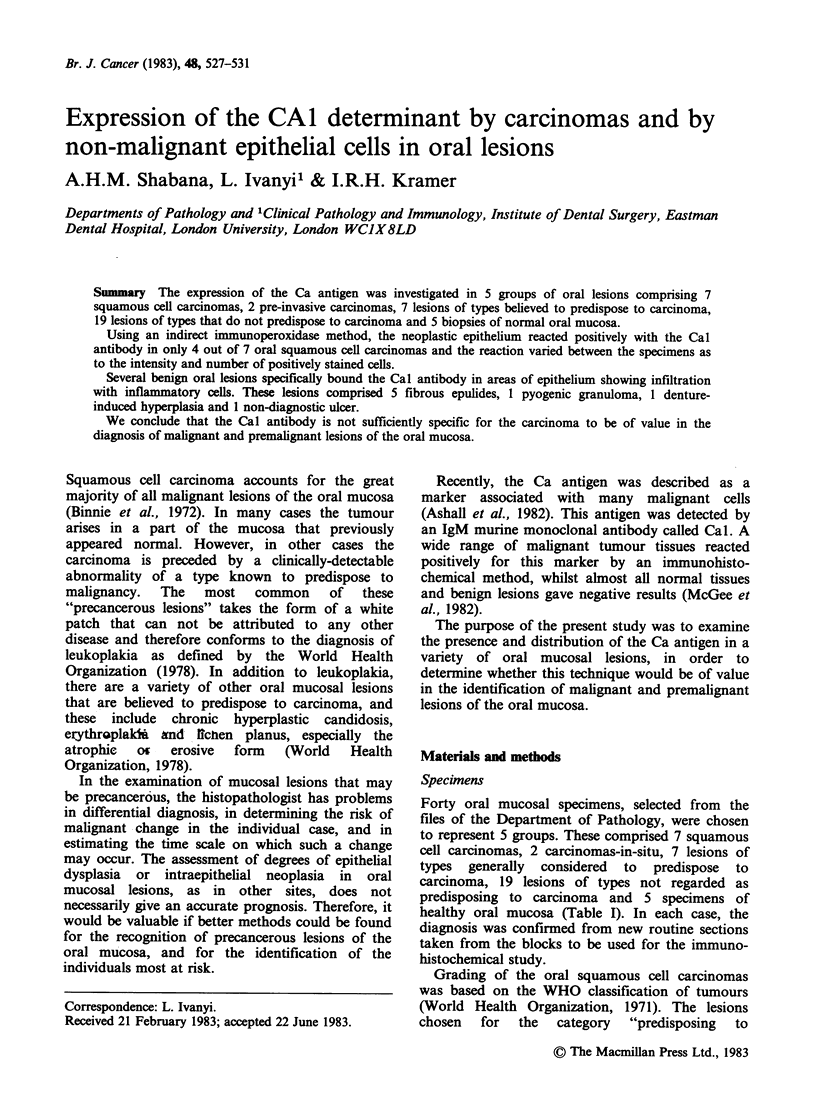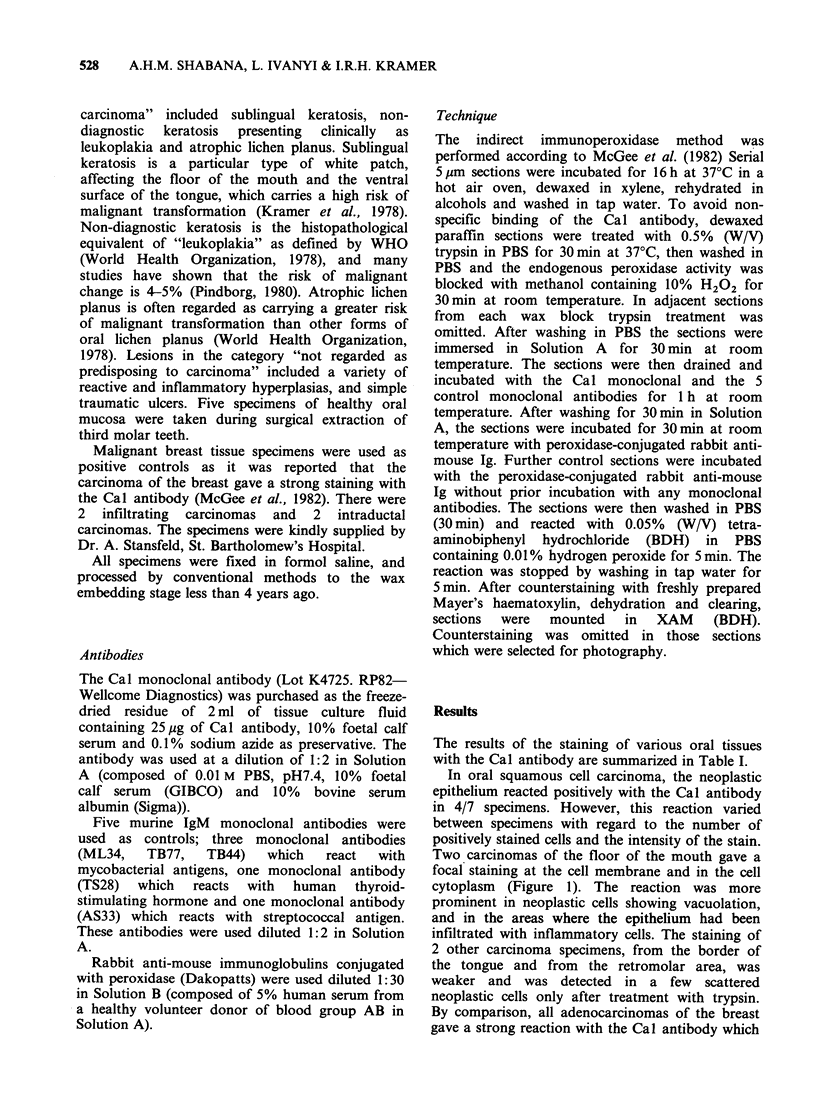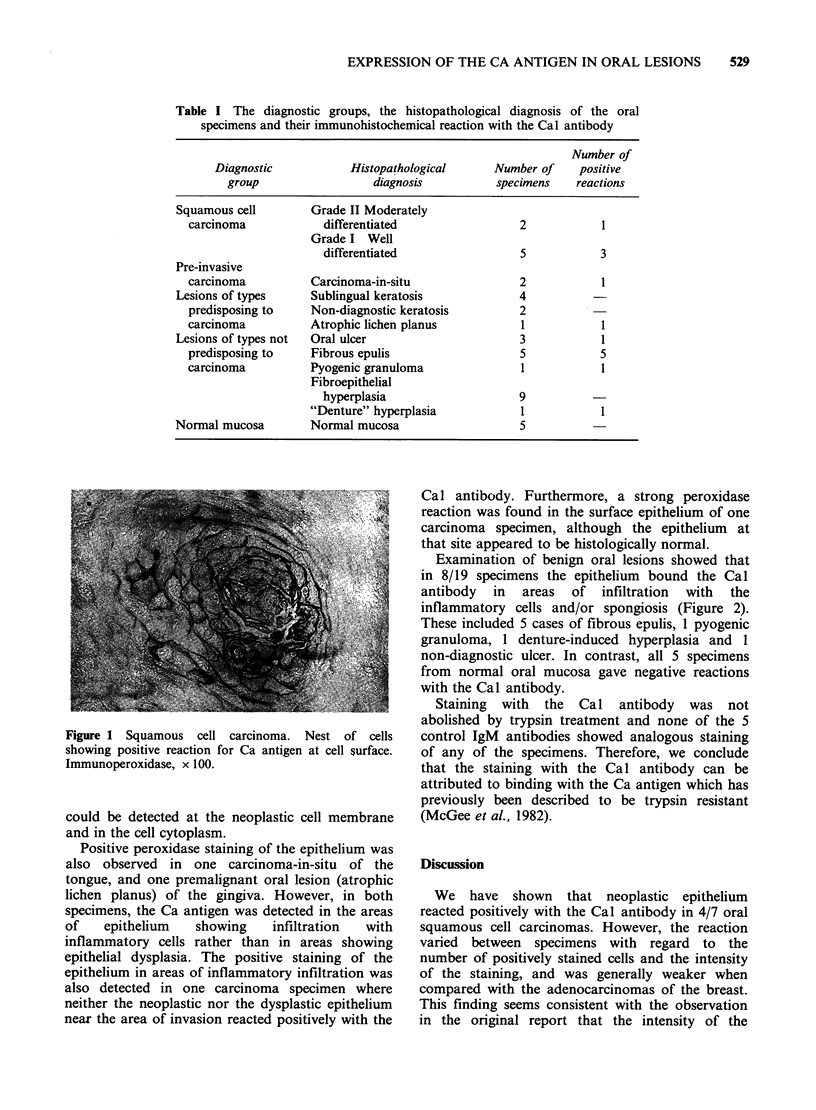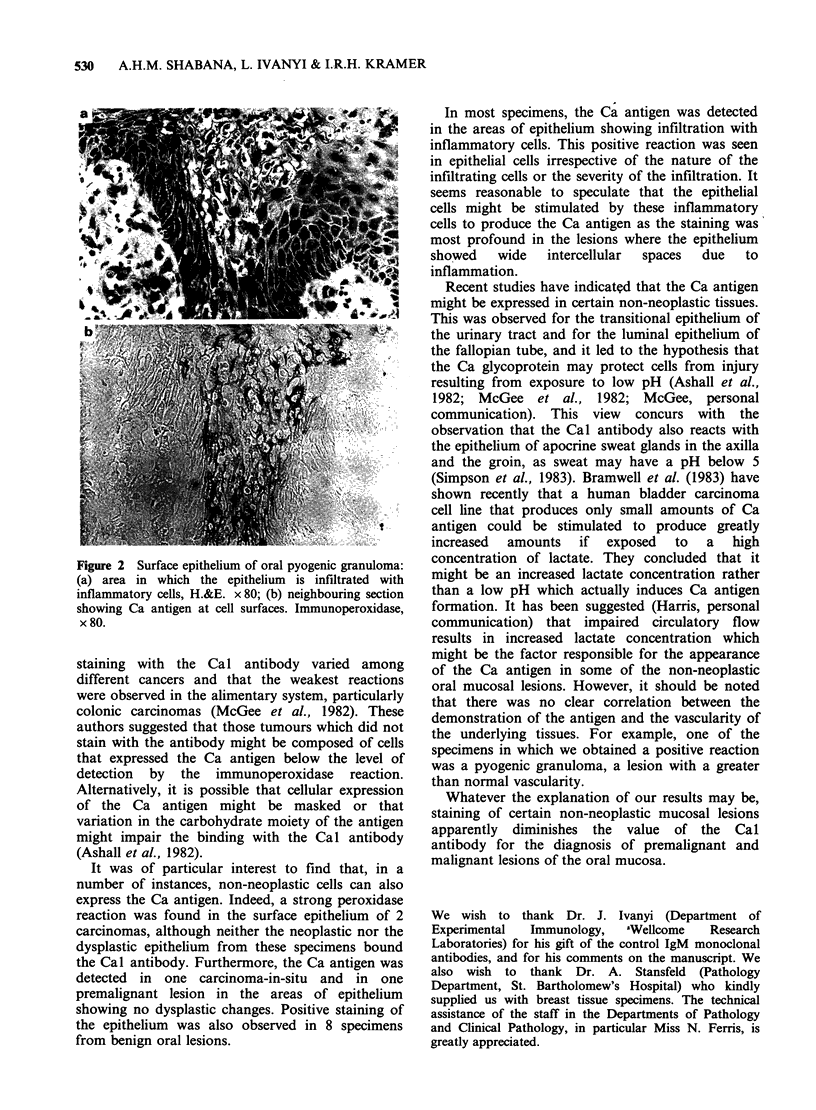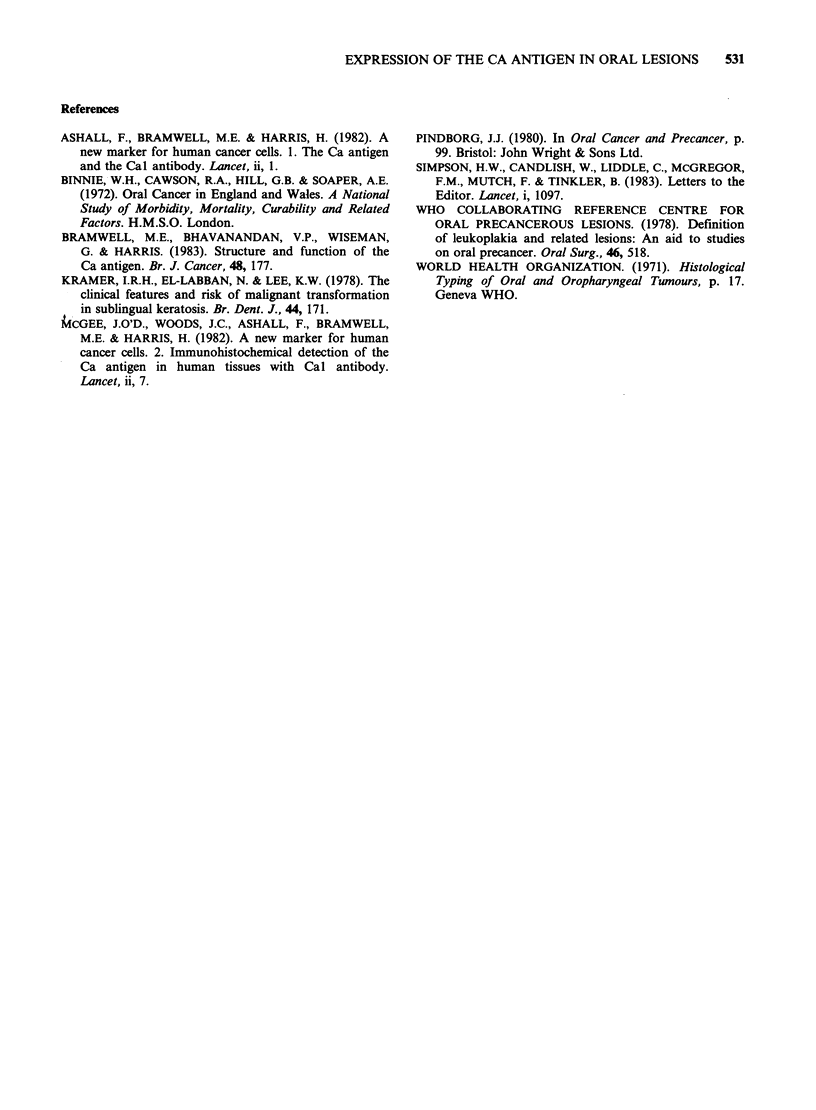# Expression of the CA1 determinant by carcinomas and by non-malignant epithelial cells in oral lesions.

**DOI:** 10.1038/bjc.1983.225

**Published:** 1983-10

**Authors:** A. H. Shabana, L. Ivanyi, I. R. Kramer

## Abstract

**Images:**


*Br. J. Cancer* (1983), **48**, 527–531

### Expression of the CA1 determinant by carcinomas and by non-malignant epithelial cells in oral lesions

A.H.M. Shabana, L. Ivanyi[1] & I.R.H. Kramer

*Departments of Pathology and [1]Clinical Pathology and Immunology, Institute of Dental Surgery, Eastman Dental Hospital, London University, London WC1X 8LD*

**Summary** The expression of the Ca antigen was investigated in 5 groups of oral lesions comprising 7 squamous cell carcinomas, 2 pre-invasive carcinomas, 7 lesions of types believed to predispose to carcinoma, 19 lesions of types that do not predispose to carcinoma and 5 biopsies of normal oral mucosa.

Using an indirect immunoperoxidase method, the neoplastic epithelium reacted positively with the Ca1 antibody in only 4 out of 7 oral squamous cell carcinomas and the reaction varied between the specimens as to the intensity and number of positively stained cells.

Several benign oral lesions specifically bound the Ca1 antibody in areas of epithelium showing infiltration with inflammatory cells. These lesions comprised 5 fibrous epulides, 1 pyogenic granuloma, 1 denture-induced hyperplasia and 1 non-diagnostic ulcer.

We conclude that the Ca1 antibody is not sufficiently specific for the carcinoma to be of value in the diagnosis of malignant and premalignant lesions of the oral mucosa.

Squamous cell carcinoma accounts for the great majority of all malignant lesions of the oral mucosa (Binnie *et al.*, 1972). In many cases the tumour arises in a part of the mucosa that previously appeared normal. However, in other cases the carcinoma is preceded by a clinically-detectable abnormality of a type known to predispose to malignancy. The most common of these "precancerous lesions" takes the form of a white patch that can not be attributed to any other disease and therefore conforms to the diagnosis of leukoplakia as defined by the World Health Organization (1978). In addition to leukoplakia, there are a variety of other oral mucosal lesions that are believed to predispose to carcinoma, and these include chronic hyperplastic candidosis, erythroplakia and lichen planus, especially the atrophic or erosive form (World Health Organization, 1978).

In the examination of mucosal lesions that may be precancerous, the histopathologist has problems in differential diagnosis, in determining the risk of malignant change in the individual case, and in estimating the time scale on which such a change may occur. The assessment of degrees of epithelial dysplasia or intraepithelial neoplasia in oral mucosal lesions, as in other sites, does not necessarily give an accurate prognosis. Therefore, it would be valuable if better methods could be found for the recognition of precancerous lesions of the oral mucosa, and for the identification of the individuals most at risk.

Recently, the Ca antigen was described as a marker associated with many malignant cells (Ashall *et al.*, 1982). This antigen was detected by an IgM murine monoclonal antibody called Ca1. A wide range of malignant tumour tissues reacted positively for this marker by an immunohistochemical method, whilst almost all normal tissues and benign lesions gave negative results (McGee *et al.*, 1982).

The purpose of the present study was to examine the presence and distribution of the Ca antigen in a variety of oral mucosal lesions, in order to determine whether this technique would be of value in the identification of malignant and premalignant lesions of the oral mucosa.

### Materials and methods

### *Specimens*

Forty oral mucosal specimens, selected from the files of the Department of Pathology, were chosen to represent 5 groups. These comprised 7 squamous cell carcinomas, 2 carcinomas-in-situ, 7 lesions of types generally considered to predispose to carcinoma, 19 lesions of types not regarded as predisposing to carcinoma and 5 specimens of healthy oral mucosa (Table I). In each case, the diagnosis was confirmed from new routine sections taken from the blocks to be used for the immunohistochemical study.

Grading of the oral squamous cell carcinomas was based on the WHO classification of tumours (World Health Organization, 1971). The lesions chosen for the category "predisposing to

Correspondence: L. Ivanyi.
Received 21 February 1983; accepted 22 June 1983.

© The Macmillan Press Ltd., 1983

carcinoma" included sublingual keratosis, non-diagnostic keratosis presenting clinically as leukoplakia and atrophic lichen planus. Sublingual keratosis is a particular type of white patch, affecting the floor of the mouth and the ventral surface of the tongue, which carries a high risk of malignant transformation (Kramer *et al.*, 1978). Non-diagnostic keratosis is the histopathological equivalent of "leukoplakia" as defined by WHO (World Health Organization, 1978), and many studies have shown that the risk of malignant change is 4–5% (Pindborg, 1980). Atrophic lichen planus is often regarded as carrying a greater risk of malignant transformation than other forms of oral lichen planus (World Health Organization, 1978). Lesions in the category "not regarded as predisposing to carcinoma" included a variety of reactive and inflammatory hyperplasias, and simple traumatic ulcers. Five specimens of healthy oral mucosa were taken during surgical extraction of third molar teeth.

Malignant breast tissue specimens were used as positive controls as it was reported that the carcinoma of the breast gave a strong staining with the Ca1 antibody (McGee *et al.*, 1982). There were 2 infiltrating carcinomas and 2 intraductal carcinomas. The specimens were kindly supplied by Dr. A. Stansfeld, St. Bartholomew's Hospital.

All specimens were fixed in formol saline, and processed by conventional methods to the wax embedding stage less than 4 years ago.

### *Antibodies*

The Ca1 monoclonal antibody (Lot K4725. RP82—Wellcome Diagnostics) was purchased as the freeze-dried residue of 2 ml of tissue culture fluid containing 25 $\mu$g of Ca1 antibody, 10% foetal calf serum and 0.1% sodium azide as preservative. The antibody was used at a dilution of 1:2 in Solution A (composed of 0.01 M PBS, pH7.4, 10% foetal calf serum (GIBCO) and 10% bovine serum albumin (Sigma)).

Five murine IgM monoclonal antibodies were used as controls; three monoclonal antibodies (ML34, TB77, TB44) which react with mycobacterial antigens, one monoclonal antibody (TS28) which reacts with human thyroid-stimulating hormone and one monoclonal antibody (AS33) which reacts with streptococcal antigen. These antibodies were used diluted 1:2 in Solution A.

Rabbit anti-mouse immunoglobulins conjugated with peroxidase (Dakopatts) were used diluted 1:30 in Solution B (composed of 5% human serum from a healthy volunteer donor of blood group AB in Solution A).

### *Technique*

The indirect immunoperoxidase method was performed according to McGee *et al.* (1982) Serial 5 $\mu$m sections were incubated for 16 h at 37°C in a hot air oven, dewaxed in xylene, rehydrated in alcohols and washed in tap water. To avoid non-specific binding of the Ca1 antibody, dewaxed paraffin sections were treated with 0.5% (W/V) trypsin in PBS for 30 min at 37°C, then washed in PBS and the endogenous peroxidase activity was blocked with methanol containing 10% $H_2O_2$ for 30 min at room temperature. In adjacent sections from each wax block trypsin treatment was omitted. After washing in PBS the sections were immersed in Solution A for 30 min at room temperature. The sections were then drained and incubated with the Ca1 monoclonal and the 5 control monoclonal antibodies for 1 h at room temperature. After washing for 30 min in Solution A, the sections were incubated for 30 min at room temperature with peroxidase-conjugated rabbit anti-mouse Ig. Further control sections were incubated with the peroxidase-conjugated rabbit anti-mouse Ig without prior incubation with any monoclonal antibodies. The sections were then washed in PBS (30 min) and reacted with 0.05% (W/V) tetra-aminobiphenyl hydrochloride (BDH) in PBS containing 0.01% hydrogen peroxide for 5 min. The reaction was stopped by washing in tap water for 5 min. After counterstaining with freshly prepared Mayer's haematoxylin, dehydration and clearing, sections were mounted in XAM (BDH). Counterstaining was omitted in those sections which were selected for photography.

### Results

The results of the staining of various oral tissues with the Ca1 antibody are summarized in Table I.

In oral squamous cell carcinoma, the neoplastic epithelium reacted positively with the Ca1 antibody in 4/7 specimens. However, this reaction varied between specimens with regard to the number of positively stained cells and the intensity of the stain. Two carcinomas of the floor of the mouth gave a focal staining at the cell membrane and in the cell cytoplasm (Figure 1). The reaction was more prominent in neoplastic cells showing vacuolation, and in the areas where the epithelium had been infiltrated with inflammatory cells. The staining of 2 other carcinoma specimens, from the border of the tongue and from the retromolar area, was weaker and was detected in a few scattered neoplastic cells only after treatment with trypsin. By comparison, all adenocarcinomas of the breast gave a strong reaction with the Ca1 antibody which

**Table I** The diagnostic groups, the histopathological diagnosis of the oral specimens and their immunohistochemical reaction with the Ca1 antibody

| *Diagnostic group* | *Histopathological diagnosis* | *Number of specimens* | *Number of positive reactions* |
|---|---|---|---|
| Squamous cell carcinoma | Grade II Moderately differentiated | 2 | 1 |
| | Grade I Well differentiated | 5 | 3 |
| Pre-invasive carcinoma | Carcinoma-in-situ | 2 | 1 |
| Lesions of types predisposing to carcinoma | Sublingual keratosis | 4 | — |
| | Non-diagnostic keratosis | 2 | — |
| | Atrophic lichen planus | 1 | 1 |
| Lesions of types not predisposing to carcinoma | Oral ulcer | 3 | 1 |
| | Fibrous epulis | 5 | 5 |
| | Pyogenic granuloma | 1 | 1 |
| | Fibroepithelial hyperplasia | 9 | — |
| | "Denture" hyperplasia | 1 | 1 |
| Normal mucosa | Normal mucosa | 5 | — |

**Figure 1** Squamous cell carcinoma. Nest of cells showing positive reaction for Ca antigen at cell surface. Immunoperoxidase, ×100.

could be detected at the neoplastic cell membrane and in the cell cytoplasm.

Positive peroxidase staining of the epithelium was also observed in one carcinoma-in-situ of the tongue, and one premalignant oral lesion (atrophic lichen planus) of the gingiva. However, in both specimens, the Ca antigen was detected in the areas of epithelium showing infiltration with inflammatory cells rather than in areas showing epithelial dysplasia. The positive staining of the epithelium in areas of inflammatory infiltration was also detected in one carcinoma specimen where neither the neoplastic nor the dysplastic epithelium near the area of invasion reacted positively with the Ca1 antibody. Furthermore, a strong peroxidase reaction was found in the surface epithelium of one carcinoma specimen, although the epithelium at that site appeared to be histologically normal.

Examination of benign oral lesions showed that in 8/19 specimens the epithelium bound the Ca1 antibody in areas of infiltration with the inflammatory cells and/or spongiosis (Figure 2). These included 5 cases of fibrous epulis, 1 pyogenic granuloma, 1 denture-induced hyperplasia and 1 non-diagnostic ulcer. In contrast, all 5 specimens from normal oral mucosa gave negative reactions with the Ca1 antibody.

Staining with the Ca1 antibody was not abolished by trypsin treatment and none of the 5 control IgM antibodies showed analogous staining of any of the specimens. Therefore, we conclude that the staining with the Ca1 antibody can be attributed to binding with the Ca antigen which has previously been described to be trypsin resistant (McGee *et al.*, 1982).

### Discussion

We have shown that neoplastic epithelium reacted positively with the Ca1 antibody in 4/7 oral squamous cell carcinomas. However, the reaction varied between specimens with regard to the number of positively stained cells and the intensity of the staining, and was generally weaker when compared with the adenocarcinomas of the breast. This finding seems consistent with the observation in the original report that the intensity of the

**Figure 2** Surface epithelium of oral pyogenic granuloma: (a) area in which the epithelium is infiltrated with inflammatory cells, H.&E. ×80; (b) neighbouring section showing Ca antigen at cell surfaces. Immunoperoxidase, ×80.

staining with the Ca1 antibody varied among different cancers and that the weakest reactions were observed in the alimentary system, particularly colonic carcinomas (McGee *et al.,* 1982). These authors suggested that those tumours which did not stain with the antibody might be composed of cells that expressed the Ca antigen below the level of detection by the immunoperoxidase reaction. Alternatively, it is possible that cellular expression of the Ca antigen might be masked or that variation in the carbohydrate moiety of the antigen might impair the binding with the Ca1 antibody (Ashall *et al.,* 1982).

It was of particular interest to find that, in a number of instances, non-neoplastic cells can also express the Ca antigen. Indeed, a strong peroxidase reaction was found in the surface epithelium of 2 carcinomas, although neither the neoplastic nor the dysplastic epithelium from these specimens bound the Ca1 antibody. Furthermore, the Ca antigen was detected in one carcinoma-in-situ and in one premalignant lesion in the areas of epithelium showing no dysplastic changes. Positive staining of the epithelium was also observed in 8 specimens from benign oral lesions.

In most specimens, the Ca antigen was detected in the areas of epithelium showing infiltration with inflammatory cells. This positive reaction was seen in epithelial cells irrespective of the nature of the infiltrating cells or the severity of the infiltration. It seems reasonable to speculate that the epithelial cells might be stimulated by these inflammatory cells to produce the Ca antigen as the staining was most profound in the lesions where the epithelium showed wide intercellular spaces due to inflammation.

Recent studies have indicated that the Ca antigen might be expressed in certain non-neoplastic tissues. This was observed for the transitional epithelium of the urinary tract and for the luminal epithelium of the fallopian tube, and it led to the hypothesis that the Ca glycoprotein may protect cells from injury resulting from exposure to low pH (Ashall *et al.,* 1982; McGee *et al.,* 1982; McGee, personal communication). This view concurs with the observation that the Ca1 antibody also reacts with the epithelium of apocrine sweat glands in the axilla and the groin, as sweat may have a pH below 5 (Simpson *et al.,* 1983). Bramwell *et al.* (1983) have shown recently that a human bladder carcinoma cell line that produces only small amounts of Ca antigen could be stimulated to produce greatly increased amounts if exposed to a high concentration of lactate. They concluded that it might be an increased lactate concentration rather than a low pH which actually induces Ca antigen formation. It has been suggested (Harris, personal communication) that impaired circulatory flow results in increased lactate concentration which might be the factor responsible for the appearance of the Ca antigen in some of the non-neoplastic oral mucosal lesions. However, it should be noted that there was no clear correlation between the demonstration of the antigen and the vascularity of the underlying tissues. For example, one of the specimens in which we obtained a positive reaction was a pyogenic granuloma, a lesion with a greater than normal vascularity.

Whatever the explanation of our results may be, staining of certain non-neoplastic mucosal lesions apparently diminishes the value of the Ca1 antibody for the diagnosis of premalignant and malignant lesions of the oral mucosa.

We wish to thank Dr. J. Ivanyi (Department of Experimental Immunology, Wellcome Research Laboratories) for his gift of the control IgM monoclonal antibodies, and for his comments on the manuscript. We also wish to thank Dr. A. Stansfeld (Pathology Department, St. Bartholomew's Hospital) who kindly supplied us with breast tissue specimens. The technical assistance of the staff in the Departments of Pathology and Clinical Pathology, in particular Miss N. Ferris, is greatly appreciated.

**References**

ASHALL, F., BRAMWELL, M.E. & HARRIS, H. (1982). A new marker for human cancer cells. 1. The Ca antigen and the Ca1 antibody. *Lancet*, ii, 1.

BINNIE, W.H., CAWSON, R.A., HILL, G.B. & SOAPER, A.E. (1972). Oral Cancer in England and Wales. *A National Study of Morbidity, Mortality, Curability and Related Factors*. H.M.S.O. London.

BRAMWELL, M.E., BHAVANANDAN, V.P., WISEMAN, G. & HARRIS. (1983). Structure and function of the Ca antigen. *Br. J. Cancer*, **48**, 177.

KRAMER, I.R.H., EL-LABBAN, N. & LEE, K.W. (1978). The clinical features and risk of malignant transformation in sublingual keratosis. *Br. Dent. J.*, **44**, 171.

MCGEE, J.O'D., WOODS, J.C., ASHALL, F., BRAMWELL, M.E. & HARRIS, H. (1982). A new marker for human cancer cells. 2. Immunohistochemical detection of the Ca antigen in human tissues with Ca1 antibody. *Lancet*, ii, 7.

PINDBORG, J.J. (1980). In *Oral Cancer and Precancer*, p. 99. Bristol: John Wright & Sons Ltd.

SIMPSON, H.W., CANDLISH, W., LIDDLE, C., MCGREGOR, F.M., MUTCH, F. & TINKLER, B. (1983). Letters to the Editor. *Lancet*, i, 1097.

WHO COLLABORATING REFERENCE CENTRE FOR ORAL PRECANCEROUS LESIONS. (1978). Definition of leukoplakia and related lesions: An aid to studies on oral precancer. *Oral Surg.*, **46**, 518.

WORLD HEALTH ORGANIZATION. (1971). *Histological Typing of Oral and Oropharyngeal Tumours*, p. 17. Geneva WHO.